# Droplet digital PCR for diagnosing invasive aspergillosis in hematological malignancies: a prospective cohort study

**DOI:** 10.1128/jcm.01140-26

**Published:** 2026-10-27

**Authors:** Roni Bitterman, Chun Fai Ng, Simran Arora, Milena Casciato, Ursula Magyar, Sophia Salazar, Naveen Usman, Zohair Pervez, Farwa Rehan, Laura N. Walti, Jonas Mattsson, Vikas Gupta, Tony Mazzulli, Shahid Husain

**Affiliations:** 1 Transplant Infectious Diseases, Ajmera Transplant Centre, University Health Network 7989, Toronto, Canada; 2 Department of Infectious Diseases, Inselspital, Bern University Hospital, University of Bern 27210, Bern, Switzerland; 3 Messner Bone Marrow Transplant Program, Princess Margaret Cancer Centre, University Health Network 7989, Toronto, Canada; 4 Division of Medical Oncology and Hematology, Princess Margaret Cancer Centre, University Health Network 7989, Toronto, Canada; 5 Department of Laboratory Medicine and Pathology, University Health Network 7989, Toronto, Canada; University of Utah Health, Salt Lake City, Utah, USA

**Keywords:** hematologic malignancy, exhaled breath condensate, droplet digital PCR, invasive aspergillosis, *Aspergillus*

## Abstract

Our objective was to evaluate the diagnostic accuracy of *Aspergillus* droplet digital PCR (ddPCR) in exhaled breath condensate (EBC) and bronchoalveolar lavage fluid (BALF) for diagnosing invasive aspergillosis (IA), and to compare its performance with quantitative real-time PCR (qPCR) in patients with hematologic malignancies. We conducted a prospective single-center study, including adults admitted to leukemia and allogeneic hematopoietic stem cell transplant units who underwent bronchoscopy for pulmonary infiltrates between October 2021 and December 2024. EBC and BALF were collected concurrently. ddPCR was performed on EBC and BALF (supernatant and cell pellet); qPCR was performed on BALF samples. IA was classified according to EORTC/MSGERC criteria without incorporating PCR into the reference standard. Diagnostic performance characteristics, including area under the receiver operating characteristic curve (AUC-ROC), were calculated. Among 203 participants, 26 (12.8%) were diagnosed with probable or proven IA. EBC ddPCR showed poor performance (AUC-ROC 0.55, 95% CI 0.43–0.67). BALF supernatant ddPCR demonstrated the best performance (AUC-ROC 0.88, 95% CI 0.80–0.96), with sensitivity of 0.65 (95% CI 0.43–0.84) and specificity of 0.96 (95% CI 0.92–0.99). BALF supernatant outperformed BALF cell pellet testing for both ddPCR and qPCR. Compared with qPCR, ddPCR showed higher sensitivity and similarly high specificity. Among participants without prior mold-active antifungal therapy, BALF supernatant ddPCR showed improved performance (AUC-ROC 0.95, 95% CI 0.89–1.00). In this large prospective study, EBC ddPCR demonstrated limited utility for the diagnosis of IA. BALF supernatant was the optimal specimen type, and ddPCR outperformed qPCR. Diagnostic performance improved substantially without prior mold-active antifungal exposure.

**IMPORTANCE:**

Invasive aspergillosis is difficult to diagnose in patients with hematologic malignancies, and non-invasive diagnostic approaches are needed. In this prospective study of 203 patients, *Aspergillus* droplet digital PCR performed poorly in exhaled breath condensate (area under the receiver operating characteristic curve 0.55; 95% CI 0.43–0.67) but showed good diagnostic performance in bronchoalveolar lavage supernatant fluid (area under the receiver operating characteristic curve 0.88; 95% CI 0.80–0.96). Droplet digital PCR demonstrated higher sensitivity than quantitative PCR with similarly high specificity, and bronchoalveolar lavage supernatant fluid consistently outperformed cell pellet testing. Among patients without prior mold-active antifungal therapy, the area under the receiver operating characteristic curve was 0.95 (95% CI 0.89–1.00). These findings support bronchoalveolar lavage supernatant fluid as the preferred specimen for *Aspergillus* droplet digital PCR and warrant further evaluation of this approach for diagnosing invasive aspergillosis.

## INTRODUCTION

Despite the changing epidemiology of invasive fungal infections with the widespread use of mold-active antifungal prophylaxis, invasive aspergillosis (IA) remains the most common invasive fungal infection in patients with hematologic malignancies (1–3). It is also associated with persistently high mortality despite advances in diagnostics and antifungal therapy (1, 4–6). Early diagnosis is important, and clinical guidelines emphasize prompt initiation of antifungal therapy in patients with IA (7, 8).

Current diagnostic methods for IA remain suboptimal. Numerous studies have evaluated the performance of galactomannan (GM) and *Aspergillus* polymerase chain reaction (PCR) in bronchoalveolar lavage fluid (BALF) and blood. In BALF, GM demonstrates a sensitivity of approximately 75%–86% and specificity of 92%–100% (9–11), while *Aspergillus* PCR shows more variable sensitivity (57%–91%) but similarly high specificity (92%–99%) (9, 11–13). In contrast, blood biomarkers generally have lower sensitivity, with serum GM around 76% (14) and blood *Aspergillus* PCR ranging from 57% to 84% (13). The diagnostic utility of serum beta-D-glucan for IA is less clear, with substantial variability reported across studies (15). Collectively, these limitations highlight the need for improved diagnostic approaches, particularly those based on non-invasive sampling, which may significantly enhance the early detection of IA.

Several studies have explored the use of exhaled breath condensate (EBC) in fungal diagnostics, with variable results (16–18). These investigations have primarily focused on the detection of GM or fungal-associated volatile organic compounds. We have previously reported on PCR-based detection of *Aspergillus* in EBC as a surveillance tool in patients with leukemia, rather than for the diagnosis of IA in those with pulmonary infiltrates (19). In that context, the approach demonstrated promising performance.

Droplet digital PCR (ddPCR) may offer several advantages over conventional PCR assays for fungal diagnostics, including enhanced sensitivity, absolute quantification without the need for standard curves, and improved tolerance to PCR inhibitors, which may be particularly relevant in low-load clinical specimens, such as EBC (20).

We aimed to evaluate the diagnostic accuracy of *Aspergillus* ddPCR in EBC and BALF for the diagnosis of IA and to compare it with that of quantitative real-time PCR (qPCR).

## MATERIALS AND METHODS

### Study design

We conducted a prospective, single-center study from October 2021 to December 2024. Consecutive adults hospitalized in the leukemia or allogeneic hematopoietic stem cell transplant (allo-HSCT) units at the Princess Margaret Cancer Center in Toronto who underwent bronchoscopy for evaluation of pulmonary infiltrates were eligible for inclusion. The study was approved by the Institutional Research Ethics Board (REB#21-5321), and all participants provided written informed consent.

### Antifungal prophylaxis

At our center, routine antifungal prophylaxis for patients with acute leukemia receiving intensive chemotherapy consists of fluconazole. Patients receiving targeted therapies (e.g., venetoclax, midostaurin, or gilteritinib) or acute lymphoblastic leukemia protocols that include vincristine are prescribed an echinocandin due to drug-drug interactions with azoles.

Patients undergoing allo-HSCT receive an echinocandin during conditioning, followed by posaconazole until day 100 post-transplant. Patients who develop graft-versus-host disease are also maintained on posaconazole.

### Study procedures

A detailed description of all laboratory procedures is provided in the Supplemental material.

### EBC and BALF collection and processing

EBC was collected using the Rtube device (Respiratory Research, Austin, USA) within 48 h of clinically indicated bronchoscopy and stored at −80°C until analysis.

BALF was obtained during bronchoscopy as part of routine clinical care. When sufficient BALF was available, an additional aliquot (approximately 5 mL) was collected for research purposes. Samples were centrifuged at 1,500 rpm for 10 min at 4°C to separate the acellular supernatant from the cellular pellet. The supernatant was aliquoted and frozen, and the cell pellet was resuspended in fetal bovine serum (Gibco, Waltham, USA) containing 10% dimethyl sulfoxide (Fisher Scientific, Waltham, USA).

### DNA extraction

Fungal DNA was extracted from EBC using the Fungi/Yeast Genomic DNA Isolation Kit© (Norgen Biotek Corp, Thorold, Canada) and eluted in 50 µL of nuclease-free water (ddH₂O). DNA from 1,000 µL BALF supernatant was extracted using the QIAamp MinElute Virus Vacuum Kit (Qiagen, Hilden, Germany). DNA from 250 µL of BALF cell pellet was extracted using the DNeasy PowerSoil Pro Kit (Qiagen) according to the manufacturer’s bead-beating protocol with minor modifications. DNA from both fractions was eluted in 50 µL of ddH_2_O.

### ddPCR in EBC and BALF

A previously described pan-*Aspergillus* hydrolysis probe assay targeting 18S rDNA was used for ddPCR on DNA extracted from EBC, BALF supernatant, and BALF cell-pellet fractions (21). Primers and probes were synthesized by Integrated DNA Technologies (IDT, Coralville, USA). Reactions were prepared using ddPCR Supermix for Probes (No dUTP) (Bio-Rad, Hercules, USA) and processed on the QX200 Droplet Digital PCR System. Droplet fluorescence was analyzed using the manufacturer’s software, and results were reported as copies per 20 µL reaction. All samples were run in duplicate with positive and negative controls.

### qPCR in BALF

For comparison, qPCR was performed on the same BALF DNA extracts using the identical pan-*Aspergillus* primer-probe set and SsoAdvanced Universal Probes Supermix on a QuantStudio 3 Real-Time PCR System (Applied Biosystems, Waltham, USA). All reactions were run in duplicate alongside the same positive and negative controls used for ddPCR.

### Outcome

The primary outcome was probable or proven IA diagnosed after the index bronchoscopy. IA was defined according to the revised criteria of the European Organization for Research and Treatment of Cancer/Mycoses Study Group Education and Research Consortium (EORTC/MSGERC), with classification based on prospective chart review (22).

As part of standard clinical care, BALF samples were submitted for fungal staining, culture, and GM testing, and serum GM was also obtained. GM was tested using an immuno-enzymatic sandwich microplate assay (Platelia, Bio-Rad, Hercules, CA), with an assay positivity cut-off of 0.5; however, results were interpreted within the EORTC/MSGERC framework described above for the classification of IA. These results contributed to the reference standard used for outcome classification. The results of ddPCR and qPCR assays were not available to treating clinicians and were not incorporated into the definition of IA.

### Statistical analysis

Cohort characteristics were summarized using counts and percentages for categorical variables and medians with interquartile ranges (IQRs) for continuous variables. Descriptive statistics were used to compare participants with and without IA.

Optimal thresholds for positivity of *Aspergillus* ddPCR in EBC, BALF supernatant, and BALF cell pellet were determined using receiver operating characteristic (ROC) curve analysis, with selection based on Youden’s index (sensitivity + specificity − 1). We also explored thresholds prioritizing sensitivity (≥0.90) and specificity (≥0.90). The area under the ROC curve (AUC) with corresponding 95% confidence intervals (CIs) was calculated for each PCR assay. The threshold for BALF *Aspergillus* qPCR (supernatant and cell pellet) was defined as a quantification cycle ≤35, based on prior literature (23).

Sensitivity, specificity, positive predictive value (PPV), negative predictive value (NPV), positive likelihood ratio (+LR), and negative likelihood ratio (–LR) were calculated for each assay (EBC ddPCR; BALF supernatant ddPCR; BALF cell pellet ddPCR; combined BALF supernatant and cell pellet ddPCR; BALF supernatant qPCR; BALF cell pellet qPCR; and combined BALF supernatant and cell pellet qPCR). Diagnostic performance analyses were calculated for each assay based on participants with available test results, with assay-specific denominators.

Sensitivity analyses were performed by restricting the cohort to participants who had not received mold-active antifungal therapy within 7 days prior to respiratory sampling. We also evaluated the diagnostic performance of EBC ddPCR among samples with condensate volume ≥0.5 mL.

All analyses were conducted using R (version 4.5.1; R Foundation for Statistical Computing, Vienna, Austria).

## RESULTS

### Study cohort

**Fig 1:**
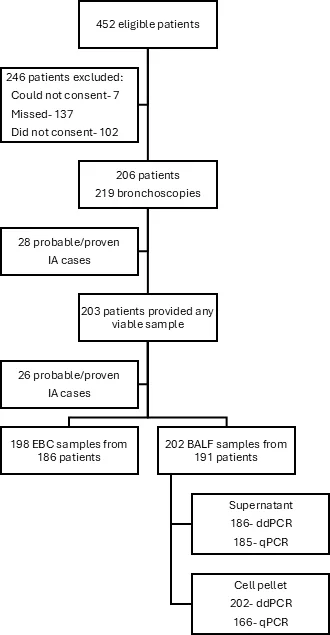
Cohort diagram. IA, invasive aspergillosis; EBC, exhaled breath condensate; BALF, bronchoalveolar lavage fluid; ddPCR, droplet digital PCR; qPCR, quantitative real-time PCR.

During the study period, 452 patients were eligible for inclusion. Of these, 206 consented to enrollment and underwent 219 bronchoscopies (Fig. 1). Three patients did not provide any viable respiratory sample (EBC or BALF) and were excluded from analyses. Among the remaining 203 participants, 186 provided 198 viable EBC specimens (>0 mL) and 191 provided 202 BALF samples. Of the 202 BALF samples, supernatant ddPCR results were available for 186 (92.1%), supernatant qPCR results for 185 (91.6%), cell pellet ddPCR results for all 202 (100.0%), and cell pellet qPCR results for 166 (82.2%), with differences in availability across testing modalities primarily due to insufficient sample volume to perform all planned analyses.

The median age of participants was 63.00 years (IQR 52.00–69.00), and 67.0% (136/203) were male (Table 1). Acute myeloid leukemia was the most common underlying hematologic malignancy (75.4%, 153/203), and 29.6% (60/203) had previously undergone allo-HSCT. Most participants were receiving chemotherapy at the time of bronchoscopy (69.0%, 140/203), most commonly induction or re-induction chemotherapy (49.3%, 69/140).

**TABLE 1:** Cohort characteristics^*f*^

Characteristic	Overall *N* = 203	No probable/proven IA *N* = 177	Probable/proven IA *N* = 26	*P* value
Age, yr, median (IQR)	63.00 (52.00, 69.00)	62.00 (52.00, 69.00)	66.50 (59.00, 71.00)	0.060
Sex, *n* (%)				0.90
Female	67 (33.0%)	58 (32.8%)	9 (34.6%)	
Male	136 (67.0%)	119 (67.2%)	17 (65.4%)	
Hematologic malignancy, *n* (%)				0.094
AML	153 (75.4%)	134 (75.7%)	19 (73.1%)	
ALL	21 (10.3%)	20 (11.3%)	1 (3.8%)	
MPAL	3 (1.5%)	2 (1.1%)	1 (3.8%)	
Lymphoma	4 (2.0%)	4 (2.3%)	0 (0.0%)	
MDS	10 (4.9%)	6 (3.4%)	4 (15.4%)	
Other^*a*^	12 (5.9%)	11 (6.2%)	1 (3.8%)	
Days from diagnosis to enrollment, median (IQR)	113.00 (19.00, 481.00)	91.00 (16.00, 457.50)	244.50 (48.00, 667.00)	0.030
Remission, *n* (%)				0.053
No	133 (65.8%)	119 (67.6%)	14 (53.8%)	
Yes	62 (30.7%)	53 (30.1%)	9 (34.6%)	
Unknown	7 (3.5%)	4 (2.3%)	3 (11.5%)	
Type of chemotherapy, *n* (%)				0.20
Induction/re-induction	69 (34.0%)	58 (32.8%)	11 (42.3%)	
Consolidation/intensification	11 (5.4%)	10 (5.6%)	1 (3.8%)	
Salvage	31 (15.3%)	27 (15.3%)	4 (15.4%)	
Maintenance	10 (4.9%)	8 (4.5%)	1 (3.8%)	
Conditioning	15 (7.4%)	11 (6.2%)	4 (15.4%)	
Other^*b*^	4 (2.0%)	4 (2.3%)	0 (0.0%)	
No chemotherapy	63 (31.0%)	59 (33.3%)	4 (15.4%)	
Prior hematopoietic stem cell transplantation, *n* (%)				0.14
No	140 (69.0%)	126 (71.2%)	14 (53.8%)	
Autologous	3 (1.5%)	3 (1.7%)	0 (0.0%)	
Allogeneic	60 (29.6%)	48 (27.1%)	12 (46.2%)	
GvHD, *n* (%)	18/60 (30.0%)	14/48 (29.2%)	4/12 (33.3%)	0.70
IA characteristics				
Proven IA			1 (3.8%)	
Probable IA			25 (96.2%)	
Positive histology			1 (3.8%)	
Positive culture^*c*^			6 (23.1%)	
Positive BALF GM^*d*^			20 (76.9%)	
Positive serum GM^*e*^			7 (26.9%)	

^*a*^ Aplastic anemia (2); mast cell leukemia (1); primary myelofibrosis (5); chronic myelomonocytic leukemia (1); prolymphocytic leukemia (2); MENGIE (metabolic disorder, 1).

^*b*^ Eltrombopag for aplastic anemia (1); chemotherapy for lymphoma (1); chemotherapy for lung cancer (1); ruxolitinib for primary myelofibrosis.

^*c*^ Four patients had growth of *Aspergillus fumigatus* and two had growth of *Aspergillus niger*.

^*d*^ All patients had a BALF GM ≥1.

^*e*^ One patient had serum GM of 0.9, the rest had ≥1.

^*f*^ IA, invasive aspergillosis; yr, years; IQR, interquartile range; AML, acute myeloid leukemia; ALL, acute lymphoblastic leukemia; MPAL, mixed phenotypic acute leukemia; MDS, myelodysplastic syndrome; GvHD, graft versus host disease; BALF, bronchoalveolar lavage fluid; GM, galactomannan.

Probable or proven IA was diagnosed in 26 participants (12.8%), including 25 (96.2%) with probable IA and 1 (3.8%) with proven IA. Diagnosis was primarily based on BALF GM positivity (index ≥ 1), which was present in 20/26 cases (76.9%). Six participants (23.1%) had a positive culture: 4 *Aspergillus fumigatus* and 2 *A. niger*. Participants with IA had a longer interval from hematologic malignancy diagnosis to bronchoscopy compared to those without IA (median 244.50 days [IQR 48.00–667.00] vs 91.00 days [IQR 16.00–457.50], *P* = 0.030).

### ROC curves and threshold analysis for ddPCR

**Fig 2:**
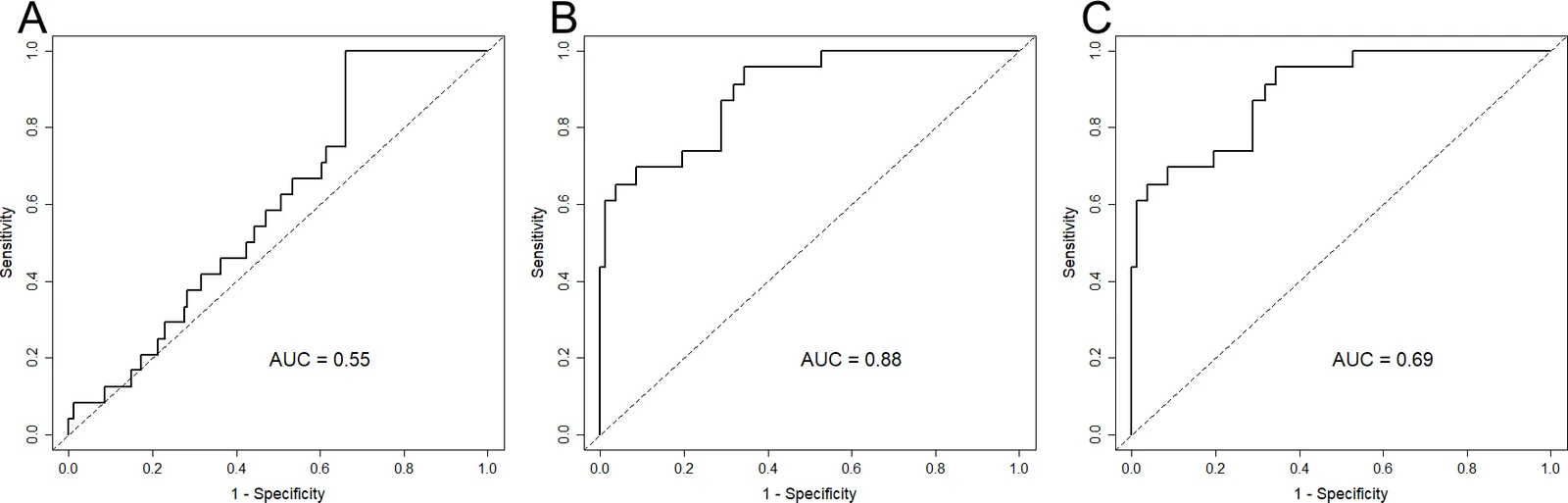
Receiver operating characteristics curves for ddPCR tests. (**A**) ROC curve for EBC *Aspergillus* ddPCR. (**B**) ROC curve for BALF supernatant *Aspergillus* ddPCR. (**C**) ROC curve for BALF cell pellet *Aspergillus* ddPCR. ROC, receiver operating characteristics; EBC, exhaled breath condensate; BALF, bronchoalveolar lavage fluid; ddPCR, droplet digital PCR.

The ROC curve for EBC ddPCR demonstrated poor discriminatory ability, with an AUC of 0.55 (95% CI 0.43–0.67). The optimal positivity threshold based on Youden’s index was 0.63 copies/20 µL. BALF supernatant ddPCR demonstrated good discriminatory ability, with an AUC of 0.88 (95% CI 0.80–0.96) and an optimal positivity threshold of 6.14 copies/20 µL. In contrast, BALF cell pellet ddPCR demonstrated only modest discriminatory ability, with an AUC of 0.69 (95% CI 0.56–0.82) and an optimal positivity threshold of 3.12 copies/20 µL (Fig. 2A through C). The AUCs for BALF supernatant and cell pellet qPCR were 0.77 (95% CI 0.67–0.87) and 0.58 (95% CI 0.49–0.67), respectively.

### Diagnostic performance of ddPCR and qPCR

EBC ddPCR demonstrated limited diagnostic performance, with moderate sensitivity (0.67, 95% CI 0.45–0.84) and poor specificity (0.45, 95% CI 0.37–0.53). In BALF, supernatant testing consistently outperformed cell pellet testing for both ddPCR and qPCR assays. Among ddPCR assays, BALF supernatant ddPCR demonstrated the best overall performance, with sensitivity of 0.65 (95% CI 0.43–0.84) and specificity of 0.96 (95% CI 0.92–0.99). In contrast, BALF cell pellet ddPCR showed substantially lower sensitivity (0.42, 95% CI 0.22–0.63). A similar pattern was observed for qPCR, where BALF supernatant qPCR outperformed cell pellet qPCR, with higher sensitivity (0.57 vs 0.17) while maintaining high specificity (0.98 vs 1.00) (Table 2).

**TABLE 2:** Diagnostic performance of *Aspergillus* ddPCR and qPCR assays for invasive aspergillosis^*a*^

	*N* samples	Test positive	IA cases	Performance (95% CI)					
				Sensitivity	Specificity	PPV	NPV	+LR	−LR
EBC *Aspergillus* ddPCR	198	112	24	0.67 (0.45–0.84)	0.45 (0.37–0.53)	0.14 (0.08–0.22)	0.91 (0.82–0.96)	1.21 (0.88–1.65)	0.74 (0.41–1.34)
BALF supernatant *Aspergillus* ddPCR	186	21	23	0.65 (0.43–0.84)	0.96 (0.92–0.99)	0.71 (0.48–0.89)	0.95 (0.91–0.98)	17.72 (7.65–41.04)	0.36 (0.21–0.63)
BALF cell pellet *Aspergillus* ddPCR	202	24	24	0.42 (0.22–0.63)	0.92 (0.87–0.96)	0.42 (0.22–0.63)	0.92 (0.87–0.96)	5.33 (2.67–10.63)	0.63 (0.45–0.89)
BALF supernatant and cell pellet *Aspergillus* ddPCR	202	37	24	0.71 (0.49–0.87)	0.89 (0.83–0.93)	0.46 (0.29–0.63)	0.96 (0.91–0.98)	6.30 (3.88–10.25)	0.33 (0.18–0.61)
BALF supernatant *Aspergillus* qPCR	185	17	23	0.57 (0.34–0.77)	0.98 (0.94–0.99)	0.76 (0.50–0.93)	0.94 (0.89–0.97)	22.89 (8.16–64.25)	0.45 (0.28–0.71)
BALF cell pellet *Aspergillus* qPCR	166	3	18	0.17 (0.04–0.41)	1.00 (0.98–1.00)	1.00 (0.29–1.00)	0.91 (0.85–0.95)	Infinite	0.83 (0.68–1.02)
BALF supernatant and cell pellet *Aspergillus* qPCR	197	17	23	0.57 (0.34–0.77)	0.98 (0.94–0.99)	0.76 (0.50–0.93)	0.94 (0.90–0.97)	24.59 (8.75–69.07)	0.45 (0.28–0.71)

^*a*^ Thresholds used (copies/20 µL for ddPCR and quantification cycle for qPCR): EBC ddPCR- ≥0.63; BALF supernatant ddPCR- ≥6.14; BALF cell pellet ddPCR- ≥3.12; BALF qPCR- ≤35. IA, invasive aspergillosis; PPV, positive predictive value; NPV, negative predictive value; +LR, positive likelihood ratio; −LR, negative likelihood ratio; EBC, exhaled breath condensate; BALF, bronchoalveolar lavage fluid; ddPCR, droplet digital PCR; qPCR, quantitative real-time PCR.

Compared with qPCR, ddPCR generally demonstrated superior diagnostic performance, particularly in BALF supernatant samples, with higher sensitivity (0.65 vs 0.57) while maintaining similarly high specificity (0.96 vs 0.98). Combining BALF supernatant and cell pellet results provided only modest incremental benefit over supernatant testing alone. For ddPCR, combined testing increased sensitivity from 0.65 to 0.70 but reduced specificity from 0.96 to 0.89. For qPCR, combined testing yielded identical performance characteristics to BALF supernatant alone (Table 2).

Overall, EBC demonstrated a high false-positive rate (1-specificity) of 0.55, compared with 0.00–0.11 for qPCR or ddPCR performed on BALF specimens.

Using alternative ddPCR thresholds, a sensitivity-prioritizing cutoff could only be meaningfully applied to BALF supernatant, yielding a sensitivity of 0.91 (95% CI 0.72–0.99) and specificity of 0.66 (95% CI 0.58–0.73) (Table S1). In contrast, specificity-prioritizing thresholds resulted in very low sensitivity for EBC ddPCR and BALF cell pellet ddPCR (0.12 [95% CI 0.03–0.32] and 0.42 [95% CI 0.22–0.63], respectively). For BALF supernatant ddPCR, a higher specificity threshold (3.91 copies/20 µL) yielded a sensitivity of 0.70 (95% CI 0.47–0.87) and a specificity of 0.90 (95% CI 0.85–0.94) (Table S2).

### Sensitivity analyses

**Fig 3:**
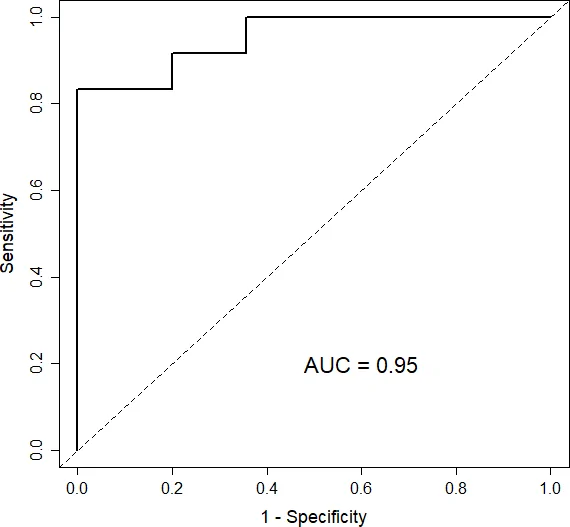
Receiver operating characteristics curves for BALF supernatant ddPCR tests in patients without mold-active antifungal therapy prior to sampling. BALF, bronchoalveolar lavage fluid; ddPCR, droplet digital PCR.

Restricting the cohort to participants who had not received mold-active antifungal therapy in the 7 days prior to respiratory sampling yielded a similar pattern of diagnostic performance to the primary analysis (Table 3). However, overall performance improved for BALF supernatant assays, with an AUC of 0.95 (95% CI 0.89–1.00; Fig. 3). In this restricted cohort, BALF supernatant ddPCR demonstrated a sensitivity of 0.83 (95% CI 0.52–0.98) with a specificity of 1.00 (0.95–1.00). BALF supernatant qPCR showed a sensitivity of 0.67 (95% CI 0.35–0.90) and a specificity of 1.00 (0.95–1.00).

**TABLE 3:** Diagnostic performance in participants without prior mold-active antifungal therapy^*a*^

	*N* samples	Test positive	IA cases	Performance (95% CI)					
				Sensitivity	Specificity	PPV	NPV	+LR	−LR
EBC *Aspergillus* ddPCR	86	48	11	0.55 (0.23–0.83)	0.44 (0.33–0.56)	0.12 (0.05–0.25)	0.87 (0.72–0.96)	0.97 (0.55–1.73)	1.03 (0.52–2.07)
BALF supernatant *Aspergillus* ddPCR	82	10	12	0.83 (0.52–0.98)	1.00 (0.95–1.00)	1.00 (0.69–1.00)	0.97 (0.90–1.00)	Infinite	0.17 (0.05–0.59)
BALF cell pellet *Aspergillus* ddPCR	89	11	13	0.38 (0.14–0.68)	0.92 (0.84–0.97)	0.45 (0.17–0.77)	0.90 (0.81–0.95)	4.87 (1.74–13.66)	0.67 (0.43–1.03)
BALF supernatant and cell pellet *Aspergillus* ddPCR	89	17	13	0.85 (0.55–0.98)	0.92 (0.84–0.97)	0.65 (0.38–0.86)	0.97 (0.90–1.00)	10.72 (4.81–23.90)	0.17 (0.05–0.60)
BALF supernatant *Aspergillus* qPCR	82	8	12	0.67 (0.35–0.90)	1.00 (0.95–1.00)	1.00 (0.63–1.00)	0.95 (0.87–0.99)	Infinite	0.33 (0.15–0.74)
BALF cell pellet *Aspergillus* qPCR	72	1	8	0.12 (0.00–0.53)	1.00 (0.94–1.00)	1.00 (0.02–1.00)	0.90 (0.81–0.96)	Infinite	0.88 (0.67–1.14)
BALF supernatant and cell pellet *Aspergillus* qPCR	87	8	12	0.67 (0.35–0.90)	1.00 (0.95–1.00)	1.00 (0.63–1.00)	0.95 (0.88–0.99)	Infinite	0.33 (0.15–0.74)

^*a*^ Thresholds used (copies/20 µL for ddPCR and quantification cycle for qPCR): EBC ddPCR- ≥0.63; BALF supernatant ddPCR- ≥6.14; BALF cell pellet ddPCR- ≥3.12; BALF qPCR- ≤35. IA, invasive aspergillosis; PPV, positive predictive value; NPV, negative predictive value; +LR, positive likelihood ratio; −LR, negative likelihood ratio; EBC, exhaled breath condensate; BALF, bronchoalveolar lavage fluid; ddPCR, droplet digital PCR; qPCR, quantitative real-time PCR.

When restricting the analysis to participants who provided ≥0.5 mL of EBC, the diagnostic performance of EBC ddPCR remained comparable to that observed in the full cohort (Table S3).

## DISCUSSION

In this prospective study of patients with acute leukemia and allo-HSCT recipients undergoing bronchoscopy for pulmonary infiltrates, we evaluated the diagnostic performance of *Aspergillus* ddPCR in EBC and BALF and compared ddPCR with conventional qPCR. We found that BALF supernatant ddPCR demonstrated the best overall diagnostic performance, with good discriminatory ability and high specificity, and consistently outperformed BALF cell pellet testing. In contrast, EBC ddPCR showed poor discriminatory ability and limited clinical utility due to low specificity and modest sensitivity. Compared with qPCR, ddPCR generally demonstrated improved sensitivity while maintaining similarly high specificity. Combining BALF supernatant and cell pellet testing increased sensitivity at the expense of reduced specificity compared with BALF supernatant testing alone. Notably, among participants who had not received mold-active antifungal therapy prior to sampling, BALF supernatant ddPCR demonstrated excellent diagnostic performance, with sensitivity increasing to 0.83 while specificity remained high (1.00).

Being a non-invasive and low-risk sampling method, EBC presents an appealing potential diagnostic approach for IA. However, in our study, EBC ddPCR demonstrated only moderate sensitivity and poor specificity, resulting in limited overall utility for either ruling in or ruling out IA. Few studies have evaluated the use of EBC for the diagnosis of IA, and the available data have been conflicting. In a study of 330 immunocompromised patients, including lung transplant recipients and patients with hematologic malignancies, EBC GM demonstrated no discriminatory ability (AUC 0.50) (18). In contrast, a study of 75 intensive care unit patients reported substantially better performance, with an AUC of 0.89 for EBC GM (24). Another study involving 133 patients reported excellent discriminatory ability (AUC 0.95) for a 47-metabolite EBC panel; however, most cases in that cohort had chronic pulmonary aspergillosis rather than IA (17). While we previously reported on the use of EBC *Aspergillus* ddPCR as a screening tool in high-risk patients (19), the present study is the first to evaluate its utility for the diagnosis of IA.

The reasons for the poor performance of EBC are uncertain, but differences in the biological characteristics and collection of EBC and BALF may have contributed. BALF directly samples the lower respiratory tract, whereas EBC provides a more indirect and potentially variable representation of respiratory secretions. Because EBC is markedly diluted by condensed water vapor (estimates of airway lining fluid dilution in EBC range from 20- to 30,000-fold) (25), the absolute amount of *Aspergillus* DNA available for extraction may frequently fall below the assay’s limit of detection, reducing sensitivity relative to BALF. EBC collection also depends on patient cooperation and may introduce greater variability in sampling, although our sensitivity analysis did not demonstrate an association between EBC volume and diagnostic performance. Conversely, EBC may be more susceptible to upper-airway contamination, which could contribute to lower specificity. These differences are unlikely to reflect laboratory processing, as EBC and BALF were frozen and subsequently analyzed using the same *Aspergillus* primer-probe set.

Few studies have directly compared BALF supernatant with cell pellet testing or evaluated ddPCR relative to qPCR. In a study including 96 patients with hematologic malignancies and suspected invasive fungal infection, BALF supernatant qPCR detected 82.0% of cases compared with 43.0% for BALF cell pellet qPCR, supporting the hypothesis that fungal DNA may be more readily detected in the acellular BALF fraction (26). Data evaluating ddPCR for IA diagnosis remain limited and heterogeneous. One study evaluating BALF ddPCR reported sensitivities of 0.67, 0.53, and 0.44 for putative, probable, and possible IA, respectively (27). Another study evaluating sputum *Aspergillus* ddPCR in 170 patients with suspected invasive fungal infection demonstrated excellent discrimination (AUC 0.97), although the use of sputum limits direct comparison with BALF-based diagnostic approaches (28). In our cohort, BALF supernatant consistently outperformed cell pellet testing for both ddPCR and qPCR. In addition, BALF supernatant ddPCR demonstrated good discriminatory ability (AUC 0.88) and higher sensitivity than qPCR while maintaining similarly high specificity. The higher sensitivity of ddPCR may relate to several analytical features of the platform. Partitioning of the sample into thousands of individual reactions can facilitate detection of low-abundance target molecules by separating rare target molecules from background material. In addition, because ddPCR relies on endpoint detection rather than quantification based on amplification kinetics, its results are less affected by differences in amplification efficiency and may be more tolerant of reaction inhibitors than qPCR. These characteristics may be particularly advantageous for BALF specimens, where fungal DNA concentrations may be low and the presence of substances that inhibit amplification may vary between samples (20).

Previous studies have consistently demonstrated that mold-active antifungal therapy prior to bronchoscopy is associated with reduced sensitivity of PCR-based BALF assays (11, 29). In a systematic review, the sensitivity of BALF qPCR decreased from 0.90 (95% CI 0.77–0.96) across all included studies to 0.58 (95% CI 0.44–0.71) in studies where mold-active treatment had been administered prior to bronchoscopy (11). Our findings reinforce this observation, as diagnostic performance improved substantially among participants who had not received mold-active antifungal therapy prior to respiratory sampling. These findings also highlight the broader diagnostic challenges associated with breakthrough invasive fungal infections. Together, these results underscore the importance of performing bronchoscopy prior to initiation of antifungal therapy whenever clinically feasible.

Our study has several limitations. First, this was a single-center study conducted at a tertiary referral cancer center with institution-specific antifungal prophylaxis practices, which may limit generalizability to other settings. Second, only 26 participants were diagnosed with IA, resulting in wide confidence intervals for some analyses. Third, a substantial proportion of participants had received mold-active antifungal therapy prior to respiratory sampling, which likely reduced fungal burden and affected assay sensitivity. However, this was explored through sensitivity analyses and reflects real-world clinical practice. Similarly, EBC volume varied across participants, although sensitivity analyses restricted to samples with higher EBC volume demonstrated similar findings. Fourth, some assay data were unavailable, particularly for BALF cell pellet qPCR analyses. Fifth, 54.4% of eligible participants were not enrolled because of logistical constraints (research staff or collection device unavailability) or participant non-consent. Although logistical factors accounted for most non-enrollments, the possibility of selection bias cannot be excluded. Finally, the ddPCR positivity thresholds identified in this study were derived internally and require external validation in independent cohorts. Validation of the assay performance against standardized or commercially available assays will also be important before clinical implementation. In addition, our pan-*Aspergillus* assay does not provide species-level identification or detection of azole resistance, which is available with some commercial platforms. Nevertheless, the large cohort, prospective design, inclusion of consecutive participants, and the fact that ddPCR and qPCR results were not incorporated into the reference standard strengthen the validity of our findings (30).

In conclusion, in this large prospective cohort of patients with hematologic malignancies undergoing bronchoscopy for pulmonary infiltrates, BALF supernatant was the optimal sample for the diagnosis of IA, and ddPCR demonstrated higher overall diagnostic performance compared to qPCR. Diagnostic performance was further enhanced in the absence of prior mold-active antifungal exposure, underscoring the importance of obtaining respiratory samples before antifungal initiation whenever clinically feasible. In contrast, EBC ddPCR showed limited clinical utility due to poor overall diagnostic performance. Prior to broader clinical implementation, the diagnostic performance and positivity thresholds of BALF ddPCR should be validated in external cohorts.

## Data Availability

Data supporting the findings of this study are available from the corresponding author upon reasonable request.

## SUPPLEMENTAL MATERIAL

Supplemental methods and tables jcm.01140-26-s0001.docx Supplemental methods and Tables S1 to S3.

ASM does not own the copyrights to Supplemental Material that may be linked to, or accessed through, an article. The authors have granted ASM a non-exclusive, world-wide license to publish the Supplemental Material files. Please contact the corresponding author directly for reuse.
