# Supplementary material for: Droplet digital PCR for diagnosing invasive aspergillosis in hematological malignancies: a prospective cohort study
Source: J Clin Microbiol. 2026 Oct 27;64(11):e01140-26. doi: 10.1128/jcm.01140-26 (PMC13753779; doi:10.1128/jcm.01140-26)
Supplement: Supplementary file 1 [file jcm.01140-26-s0001.docx]

**Supplementary material- Droplet Digital PCR for Diagnosing Invasive Aspergillosis in Hematological Malignancies: A Prospective Cohort Study**

**Table of contents**

Description of laboratory procedures………………………………………………………………page 2

Supplementary Table 1. Diagnostic performance of *Aspergillus* ddPCR using
sensitivity-prioritizing threshold………………………………………………………………………page 5

Supplementary Table 2. Diagnostic performance of *Aspergillus* ddPCR using
specificity-prioritizing threshold………………………………………………………………………page 6

Supplementary Table 3. Diagnostic performance of EBC ddPCR among samples
with EBC volume ≥0.5 mL……………………………………………………………………………….page 7

**Description of laboratory procedures**

Exhaled breath condensate (EBC) and bronchoalveolar lavage fluid (BALF) collection and processing

EBC was collected using the Rtube© device (Respiratory Research, Austin, TX), a portable, disposable system with a one-way valve designed for non-invasive collection of exhaled air. Samples were stored at -80°C. EBC collection was performed within 48 hours of bronchoscopy, though in most cases it was collected concurrently.

Bronchoscopy was performed according to routine clinical practice. BALF (approximately 5 mL) was collected for research purposes at the time of bronchoscopy. BALF was centrifuged at 1500 rpm for 10 minutes at 4°C to separate the cellular fraction from the acellular supernatant. The supernatant was carefully aspirated and aliquoted. The cell pellet was resuspended in 1 mL of fetal bovine serum (Gibco, Thermo Fisher Scientific, Waltham, MA, USA) containing 10% dimethyl sulfoxide (Fisher Scientific, part of Thermo Fisher Scientific, Waltham, MA, USA).

DNA extraction

Fungal DNA was extracted from EBC using the Fungi/Yeast Genomic DNA Isolation Kit© (Norgen Biotek Corp, Thorold, Ontario, Canada) according to the manufacturer’s instructions and eluted in 50 µL of nuclease‑free water (ddH₂O). DNA extraction from the acellular BALF fraction was performed using 1000 µL of BALF supernatant with the QIAamp MinElute Virus Vacuum Kit (Qiagen, Hilden, Germany), following the manufacturer’s protocol for viral nucleic acid purification from cell‑free body fluids. For the cellular fraction, 250 µL of the resuspended BALF cell pellet was processed using the DNeasy PowerSoil Pro Kit (Qiagen, Hilden, Germany) according to the manufacturer’s bead‑beating-based lysis protocol, with the following modifications: 200 µL of phenol:chloroform:isoamyl alcohol (25:24:1; MilliporeSigma, Burlington, MA, USA) was added immediately prior to bead beating, and the volume of Solution CD1 was reduced to 600 µL. DNA from both BALF fractions was eluted in 50 µL of ddH₂O for downstream ddPCR and qPCR analyses.

Droplet digital polymerase chain reaction (ddPCR) in EBC and BALF

A previously described set of pan-*Aspergillus* primers and probes targeting 18s rDNA and capable of detecting the full spectrum of *Aspergillus* species was used. ^1^ Primers and probes were synthesized by Integrated DNA Technologies (IDT, Coralville, IA, USA). ddPCR reactions were prepared using ddPCR Supermix for Probes (No dUTP) (Bio‑Rad, Hercules, CA, USA) with primers at a final concentration of 900 nM and probe at 250 nM in a total reaction volume of 21 µL containing 4 µL of extracted DNA template. Twenty microliters of each reaction mixture were loaded into a DG8 cartridge with droplet generation oil and emulsified using the QX200 Droplet Digital PCR System (Bio‑Rad) according to the manufacturer’s instructions.

PCR amplification was performed using a C1000-thermocycler (Bio-Rad, Hercules, CA, USA) with a ramp rate of 2°C/s under the following cycling conditions: 95°C for 10 minutes; 40 cycles of 94°C for 30 seconds and 57°C for 1 minute; followed by 98°C for 10 minutes and a hold at 4°C. Droplet fluorescence was measured using the QX200-Droplet-Reader (Bio-Rad, Hercules, CA), and results were analyzed with QuantaSoft™ software (Bio-Rad, Hercules, CA). Each sample was run in duplicate alongside a positive control consisting of DNA from *Aspergillus fumigatus* strain NRRL-163 (ATCC, Manassas, VA, USA) and a negative control consisting of ddH_2_O. Wells containing >10,000 droplets were considered acceptable, and results were reported as copies/20 µL. Samples with discordant duplicate results underwent repeat testing.

Quantitative real-time PCR (qPCR) in BALF

For comparison, qPCR was performed using the same pan‑*Aspergillus* primer-probe set (IDT, Coralville, IA, USA), with primers at a final concentration of 900 nM, probe at 250 nM, and 4 µL of extracted DNA template in a total reaction volume of 20 µL. The DNA extracts analyzed by ddPCR were used for qPCR to enable direct comparison between platforms. Reactions were prepared using SsoAdvanced Universal Probes Supermix (Bio‑Rad, Hercules, CA, USA) according to the manufacturer’s instructions for probe‑based assays.

Thermal cycling was performed on a QuantStudio 3 Real‑Time PCR System (Applied Biosystems, Thermo Fisher Scientific, Waltham, MA, USA) under the following conditions: 50°C for 2 minutes; 95°C for 10 minutes; 40 cycles of 95°C for 30 seconds and 57°C for 1 minute. All reactions were run in duplicate alongside the same positive and negative controls used for ddPCR. Quantification cycle (Cq) values were generated using Design & Analysis 2 software (Applied Biosystems, Thermo Fisher Scientific, Waltham, MA, USA). Samples with discordant duplicate results underwent repeat testing.

**Supplementary Table 1.** Diagnostic performance of *Aspergillus* ddPCR using sensitivity-prioritizing threshold.

|  | **N samples** | **IA cases** | **Performance (95% CI)** | | | | | |
| --- | --- | --- | --- | --- | --- | --- | --- | --- |
|  |  |  | **Sensitivity** | **Specificity** | **PPV** | **NPV** | **+LR** | **-LR** |
| **EBC *Aspergillus* ddPCR** | 198 | 24 | NA | NA | NA | NA | NA | NA |
| **BALF supernatant *Aspergillus* ddPCR** | 186 | 23 | 0.91 (0.72-0.99) | 0.66 (0.58-0.73) | 0.28 (0.18-0.39) | 0.98 (0.94-1.00) | 2.71 (2.11-3.47) | 0.13 (0.03-0.50) |
| **BALF cell pellet *Aspergillus* ddPCR** | 202 | 24 | NA | NA | NA | NA | NA | NA |
| **BALF supernatant and cell pellet *Aspergillus* ddPCR** | 202 | 24 | NA | NA | NA | NA | NA | NA |

Thresholds used (copies/20 µL): EBC ddPCR- no threshold had >90% sensitivity; BALF supernatant ddPCR- ≥0.76; BALF cell pellet ddPCR- no threshold had >90% sensitivity.

IA: invasive aspergillosis; PPV: positive predictive value; NPV: negative predictive value; +LR: positive likelihood ratio; -LR: negative likelihood ratio; EBC: exhaled breath condensate; BALF: bronchoalveolar lavage fluid; ddPCR: droplet digital PCR.

**Supplementary Table 2.** Diagnostic performance of *Aspergillus* ddPCR using specificity-prioritizing threshold.

|  | **N samples** | **IA cases** | **Performance (95% CI)** | | | | | |
| --- | --- | --- | --- | --- | --- | --- | --- | --- |
|  |  |  | **Sensitivity** | **Specificity** | **PPV** | **NPV** | **+LR** | **-LR** |
| **EBC *Aspergillus* ddPCR** | 198 | 24 | 0.12 (0.03-0.32) | 0.90 (0.85-0.94) | 0.15 (0.03-0.38) | 0.88 (0.83-0.93) | 1.28 (0.40-4.04) | 0.97 (0.83-1.14) |
| **BALF supernatant *Aspergillus* ddPCR** | 186 | 23 | 0.70 (0.47-0.87) | 0.90 (0.85-0.94) | 0.50 (0.32-0.68) | 0.95 (0.91-0.98) | 7.09 (4.14-12.14) | 0.34 (0.18-0.63) |
| **BALF cell pellet *Aspergillus* ddPCR** | 202 | 24 | 0.42 (0.22-0.63) | 0.90 (0.85-0.94) | 0.37 (0.19-0.58) | 0.92 (0.87-0.96) | 4.36 (2.27-8.40) | 0.64 (0.46-0.91) |
| **BALF supernatant and cell pellet *Aspergillus* ddPCR** | 202 | 24 | 0.75 (0.53-0.90) | 0.83 (0.76-0.88) | 0.37 (0.23-0.52) | 0.96 (0.92-0.99) | 4.31 (2.90-6.39) | 0.30 (0.15-0.61) |

Thresholds used (copies/20 µL): EBC ddPCR- ≥5.28; BALF supernatant ddPCR- ≥3.91; BALF cell pellet ddPCR- ≥2.91.

IA: invasive aspergillosis; PPV: positive predictive value; NPV: negative predictive value; +LR: positive likelihood ratio; -LR: negative likelihood ratio; EBC: exhaled breath condensate; BALF: bronchoalveolar lavage fluid; ddPCR: droplet digital PCR.

**Supplementary Table 3.** Diagnostic performance of EBC ddPCR among samples with EBC volume ≥0.5 mL.

|  | **N samples** | **IA cases** | **Performance (95% CI)** | | | | | |
| --- | --- | --- | --- | --- | --- | --- | --- | --- |
|  |  |  | **Sensitivity** | **Specificity** | **PPV** | **NPV** | **+LR** | **-LR** |
| **EBC *Aspergillus* ddPCR** | 163 | 21 | 0.71 (0.48-0.89) | 0.44 (0.35-0.52) | 0.16 (0.09-0.25) | 0.91 (0.82-0.97) | 1.27 (0.93-1.72) | 0.65 (0.32-1.32) |

Threshold used (copies/20 µL): EBC ddPCR- ≥0.63.

IA: invasive aspergillosis; PPV: positive predictive value; NPV: negative predictive value; +LR: positive likelihood ratio; -LR: negative likelihood ratio; EBC: exhaled breath condensate; ddPCR: droplet digital PCR.

References

1.	Fernandez-Molina J V., Abad-Diaz-de-Cerio A, Sueiro-Olivares M, et al. Rapid and specific detection of section Fumigati and Aspergillus fumigatus in human samples using a new multiplex real-time PCR. *Diagn Microbiol Infect Dis*. 2014;80(2):111-118. doi:10.1016/J.DIAGMICROBIO.2014.06.003
